# Targeting of Tumor Neovasculature with GrB/VEGF_121_, a Novel Cytotoxic Fusion Protein

**DOI:** 10.3390/biomedicines5030042

**Published:** 2017-07-17

**Authors:** Khalid A. Mohamedali, Michael G. Rosenblum

**Affiliations:** Department of Experimental Therapeutics, UT M.D. Anderson Cancer Center, Houston, TX 77030, USA; mrosenbl@mdanderson.org

**Keywords:** Granzyme B, fusion protein, angiogenesis, vascular targeting

## Abstract

Angiogenesis is a critical process in numerous diseases, and intervention in neovascularization has therapeutic value in several disease settings, including ocular diseases, arthritis, and in tumor progression and metastatic spread. Various vascular targeting agents have been developed, including those that inhibit growth factor receptor tyrosine kinases, blocking antibodies that interfere with receptor signal transduction, and strategies that trap growth factor ligands. Limited anti-tumor efficacy studies have suggested that the targeted delivery of the human pro-apoptotic molecule Granzyme B to tumor cells has significant potential for cancer treatment. Here, we review biological vascular targeting agents, and describe a unique vascular targeting agent composed of Granzyme B and the VEGF receptor ligand VEGF_121_. The fusion protein GrB/VEGF_121_ demonstrates cytotoxicity at nanomolar or sub-nanomolar levels, excellent pharmacokinetic and efficacy profiles, and has significant therapeutic potential targeting tumor vasculature.

## 1. Introduction

### 1.1. Angiogenesis and Vascular Targeting Agents in the Clinic

Neovascularization is a normal process that occurs during growth and development, and critical during processes such as wound healing. It is also an important process in several disease states, such as tumor maturation and during metastatic spread, and interfering with this process has been shown to have therapeutic benefits [1,2,3,4]. As such, various elements of tumor neovascularization have been the focus of drug development strategies, and interventions at different points have been developed with varying levels of success in pre-clinical and clinical settings. Some such strategies include: inhibiting the downstream signaling of growth factor receptor tyrosine kinases [5,6,7], the development of blocking antibodies to attenuate receptor signal transduction [8,9,10,11], entrapment of growth factor ligands to prevent receptor activation [12,13,14], and the use of vascular-targeted photodynamic therapy [15]. Vascular targeting agents have also been tested in combination with other therapies, such as radiotherapy [16].

### 1.2. VEGF Receptor Targeting to Inhibit Angiogenesis

Numerous factors have been identified as playing significant roles during tumor neovascularization. Indeed, the complexity of this process is underscored by the fact that molecules that play a role in driving critical events continue to be identified. One key factor is vascular endothelial growth factor-A (VEGF-A) which, along with its receptors, is exceptionally important in many aspects of neovascularization [7,17,18,19]. VEGFR-1 and VEGFR-2 are generally over-expressed in tumor neovasculature and normalize as the vasculature matures [20]. Indeed, of the over 1600 clinical trials on oncology interventions against pathways related to tumor vasculature reported worldwide since January 2010 (Table S1), 233 are related to VEGF, either novel targeting agents or post-anti-VEGF therapy (Table S2).

### 1.3. Development of Fusion Proteins for Targeted Therapy

Protein-based therapeutics that precisely target cell surface receptors generally incorporate ligands or antibodies as the targeting moiety. Ligand-based therapeutics include fusion proteins that utilize the receptor-binding domains of native protein ligands to guide cytotoxic payloads to internalize and kill the specifically targeted cell. On the other hand, antibody-based therapeutics, which include antibody drug conjugates (ADCs) and immunotoxins (ITs), employ the antigen recognition domains of immunoglobulin (Ig) molecules to identify the targeted cell. Since January 2010, just 65 studies with fusion proteins as interventions have been initiated in oncology. Of these, just one has reached Phase 3 or 4 (Table S3): the use of recombinant human tumor necrosis factor-α receptor II IgG Fc fusion protein injections in the treatment of active axial spondyloarthritis. Studies currently in Phase 2 include the following fusion proteins targeting EphB4: Dalantercept, which binds to ALK1 ligands; L19IL2, which contains the vascular targeting antibody L19; F16IL2, which targets the A1 domain of tenascin-C; and cancer vaccines, among others. To date, relatively few ligand-based targeted proteins have been FDA approved, the most prominent being denileukin diftitox (Ontak), a fusion protein comprising the cytokine IL-2 fused to diphtheria toxin (DT) for treatment against CD25-positive cutaneous T-cell lymphoma (CTCL) [21]. Ontak targets the IL-2 receptor (IL-2R) upregulated on tumor cells, and internalizes to deliver diphtheria toxin into the cell, triggering an apoptotic response [21]. However, as described below, there are some drawbacks to the use of non-human toxins as cytotoxic payloads.

As a targeting agent, VEGF-A itself has been utilized by numerous laboratories in recombinant growth factor fusion proteins delivering various toxins [22,23,24,25,26] to VEGF receptor-bearing target cells. Our laboratory focused on the VEGFR-targeting ability of VEGF_121_, the smallest VEGF-A isoform, and developed VEGF_121_-based fusion proteins, particularly with gelonin, a potent plant toxin that results in the irreversible inhibition of protein synthesis. VEGF_121_/rGel showed excellent efficacy in subcutaneous, xenograft, orthotopic, and experimental metastasis models. The construct targeted angiogenesis, osteoclastogenesis and bone formation, and significantly reduced overall tumor burden, as well as inhibited tumor growth [27,28,29,30,31,32,33].

One of the concerns with fusion proteins with non-human payloads has been the potential for immunogenicity. To mitigate this, studies have been undertaken to develop cytotoxic payloads with reduced immunogenicity by identifying, and modifying, regions that potentially generate an immunogenic response [34,35], which may alleviate part of the immunogenicity concern. On the other hand, targeted cytotoxic fusion proteins composed entirely of human sequences represent an attractive alternative for application as anticancer agents. Indeed, the vast majority of the fusion proteins used in clinical trials recently contain human cytotoxic effectors or payloads, with the notable non-human exception of fusion proteins containing a deimmunized form of DT.

## 2. Granzyme B as a Cytolytic Agent

### 2.1. Granzyme B Mechanism of Action

The granzyme family of serine proteases is established as a vital component of the immune system and is important in preventing viral infection and tumor development. Granzymes, along with the pore-forming protein perforin, are delivered to the target cell by cytotoxic T lymphocytes (CTLs) and natural killer cells, resulting in apoptosis induced by direct and indirect activation of caspases and damage to mitochondria [36]. The 25 kDa molecule Granzyme B (GrB) is generally considered to be the most potent member of the granzyme family, capable of inducing both caspase-dependent and caspase-independent apoptosis. Upon entry into the cytosol, GrB triggers the release of cytochrome c from mitochondria and the onset of the apoptosis cascade by activating procaspases-3, -7 and -9 or, as noted above, initiating apoptosis via caspase-independent mechanisms [37,38].

### 2.2. Advantages of GrB-Based Constructs over Other Immunotoxins or ADCs

Creation of an immunotoxin requires several factors to be taken into consideration. Among the key criteria are the potential of the therapeutic to trigger an immunogenic response. In addition, the resulting product cannot be too toxic or lack potency, and the linkers must be sufficiently stable in circulation. The development of immunotoxins composed entirely of human sequences is a significant advantage in mitigating against the possible development of immunogenicity. Bacterial and plant toxin-based immunotoxins have demonstrated remarkable potency and specificity, but a number of obstacles limit their clinical application [39,40]. For example, off-target binding to normal vasculature of toxins such as DT and ricin A chain (RTA) has resulted in vascular damage, leading to the loss of vascular integrity (vascular leak syndrome, VLS), a potentially life-threatening condition that can result in organ failure [41,42]. In addition, instances of immunogenic responses have also been identified in the case of DT as well as other bacterial and plant toxins [43], which limits the number of therapeutic regimens and their potential value in long-term treatment [44]. Immune responses to the toxins in patients also result in the rapid clearance of subsequent courses of therapy, and limit the number of treatment cycles [45,46]. Toxin immunogenicity is being addressed by engineering B-cell epitopes on the structure, but these molecules may be difficult to humanize completely [35,47,48].

There are now a number of clinically effective ADCs demonstrating remarkable activity, and many of these constructs were driven by the impressive success of the Trastuzumab-DM1 (T-DM1) conjugate [49,50]. On the other hand, there are limitations with antibody–drug conjugates, such as facile aggregation, off-target toxicity and potential development of cells with multidrug resistance (MDR) [51,52].

Granzyme B-based fusion proteins, on the other hand, exert a multi-modal and well-known mechanism of cytotoxic action. It is of significance that inhibitors of caspase activation have little impact on the overall cytotoxicity of some GrB constructs, which attests to the presence of multiple, redundant, pro-apoptotic pathways activated by this molecule [53,54,55]. Limited studies against MDR^+^ tumor cells show that the expression of MDR does not seem to result in cross-resistance to GrB-based fusion constructs, which suggests that the emergence of resistance to this class of agents may be difficult from a biological perspective.

A key advantage of Granzyme B as a cytotoxic payload is its extremely limited ability to enter the cell without a carrier (such as a targeting molecule) or via the generation of perforin-mediated pores in the cell membrane, and its relative inertness outside of the cell. In addition, off-target in vivo toxicity is limited, due to the presence of stable amino acid linkers between the targeting molecule and the cytotoxic payload. To date, studies in mice with doses as high as 40 mg/kg have demonstrated a complete lack of toxicity (Zhou, Mohamedali et al. 2014 [55]. Finally, while the upregulation of resistance mechanisms has been observed in cells in response to some chemotherapeutic agents as well as to ADCs, our studies with a limited number of GrB-based constructs have indicated that the development of this resistance does not correlate to resistance to GrB-based targeting [53,55,56,57].

Because of the many advantages of GrB, as well as to mitigate for the possible development of immunogenicity that has been observed in patients treated with ADCs and immunotoxin-based therapies, several laboratories, including ours, initiated the development of recombinant cell death-inducing fusion proteins, with human GrB as the cytotoxic payload [53,54,55,58,59,60,61,62,63,64,65]. We and others have shown that fusion constructs delivering GrB by either antibody or ligand based targeting have highly selective cytotoxic effects when delivered to the cytoplasm.

Below, we review some of the unique features, including specific cytotoxicity, mechanism of action, and in vivo studies of a unique vascular targeting agent, GrB/VEGF_121_, a novel pro-apoptotic agent targeting tumor vasculature.

## 3. Construction, Expression and Purification of GrB/VEGF_121_

**Figure 1:**
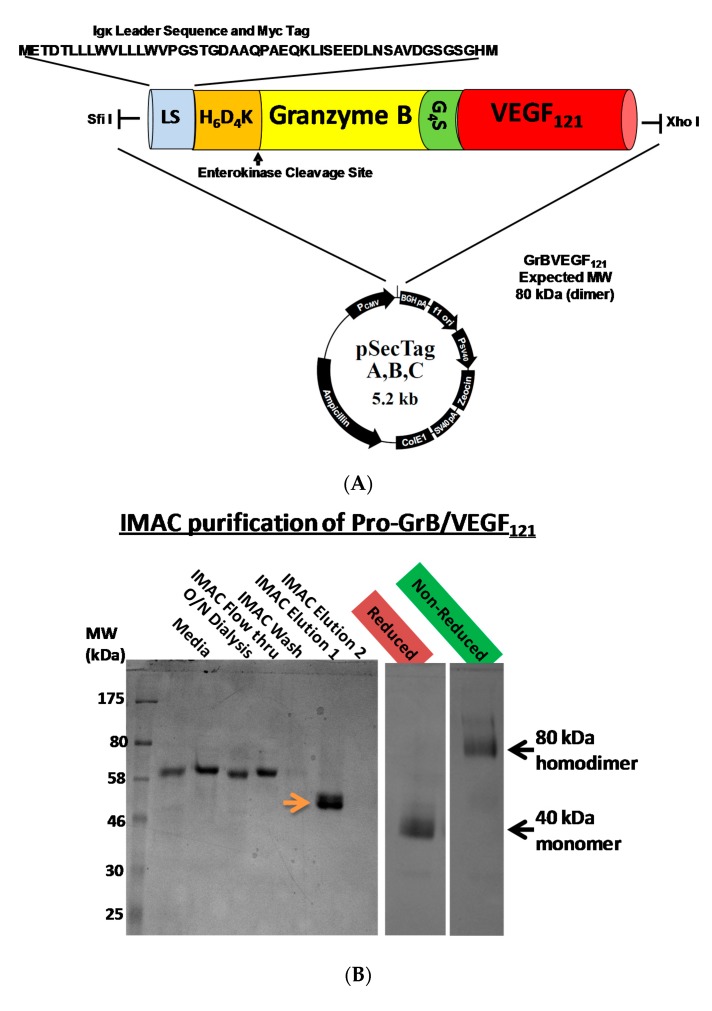

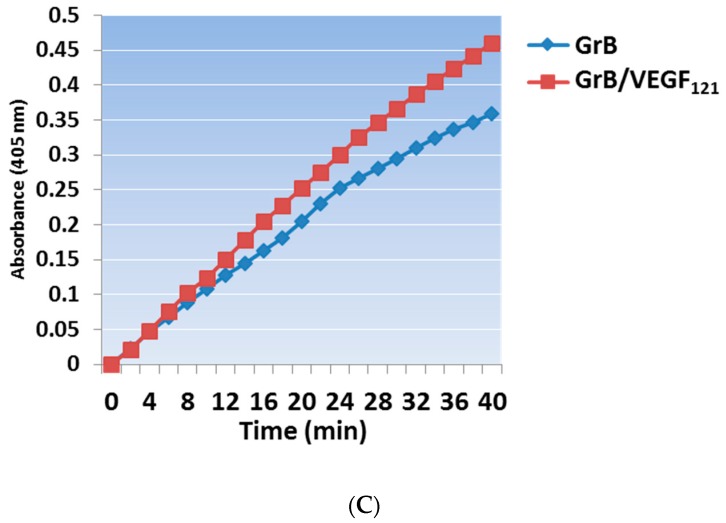
Construction, expression and purification of Granzyme B (GrB)/VEGF_121_. (**A**) The Granzyme B/VEGF_121_ cassette includes an N-terminus leader peptide for secretion of the protein into the conditioned media, followed by a His_6_-tag and a peptide sequence recognized by enterokinase. The cassette is inserted into the mammalian expression vector pSecTag. (**B**) pro-GrB/VEGF_121_, purified by metal affinity chromatography, is activated by removal of the His_6_-tag by enterokinase. (**C**) The resulting 80 kDa dimer has the equivalent enzymatic activity of free Granzyme B.

Mammalian expression of the Granzyme B/VEGF_121_ construct was initiated by fusing the GrB/VEGF_121_ DNA cassette into the pSecTag vector (Figure 1A). We have expressed GrB/VEGF_121_ using HEK-293T, HEK-293E, and CHO-S cells, and harvested the secreted fusion protein under serum-free conditions (Figure 1B). The construct is expressed as an inactive pro-GrB/VEGF_121_ form. Removal of the N-terminus histidine tag results in an 80 kDa dimer. The expressed protein is enzymatically active, as determined by a chromogenic assay measuring the rate of substrate turnover (Figure 1C).

### 3.1. In Vitro Internalization and Cytotoxic Activity

**Figure 2:**
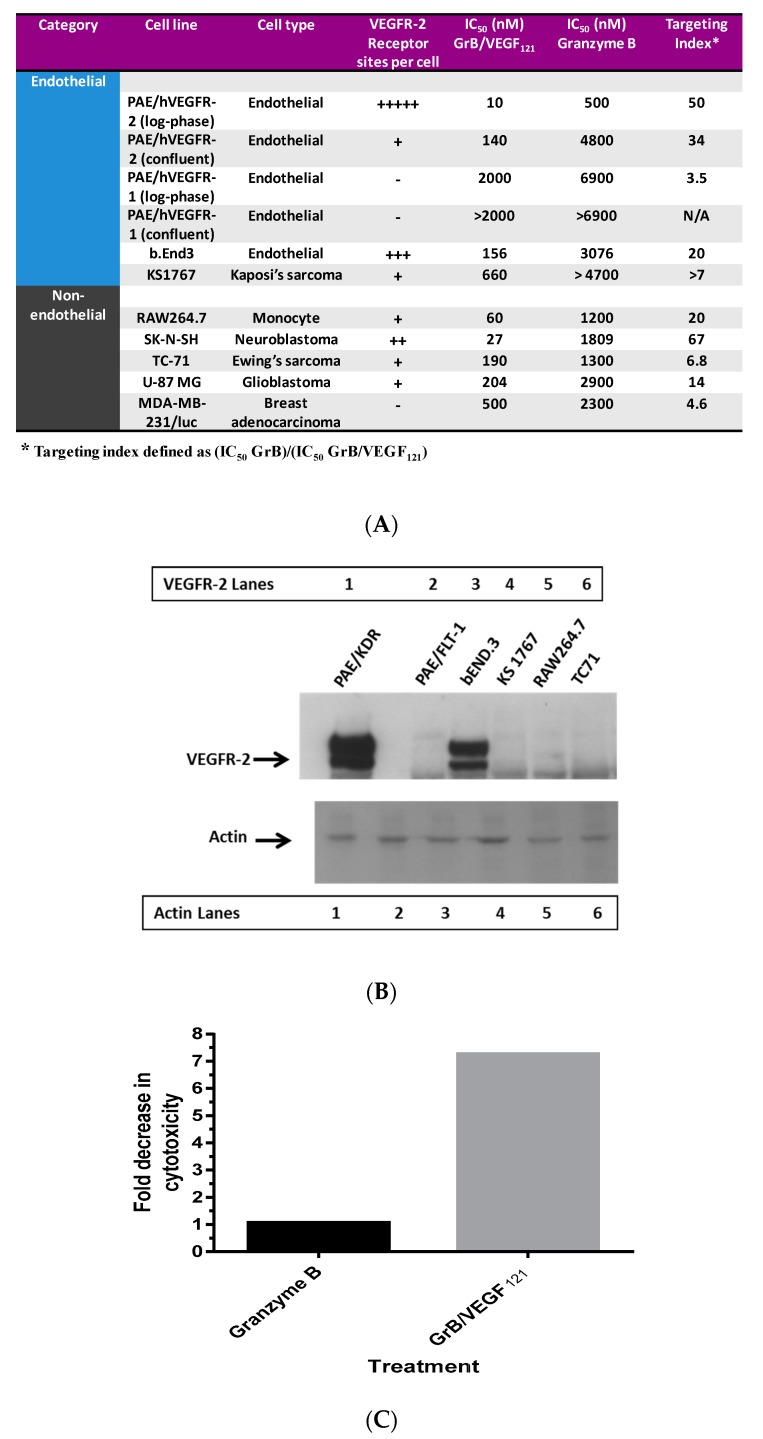
In vitro cytotoxicity of GrB/VEGF_121_. (**A**) Purified GrB/VEGF_121_ is preferentially cytotoxic to log-phase endothelial cells that over-express VEGFR-2. Other cells, such as the neuroblastoma cell line SK-N-SH and the glioblastoma cell line U87-MG, are also sensitive. (**B**) Expression levels of VEGFR-2 in some of the cell lines tested indicate some relationship between VEGFR-2 levels and sensitivity to GrB/VEGF_121_. (**C**) The impact on cytotoxicity upon pre-incubation of cells with 1 µM VEGF_121_.

**Figure 3:**
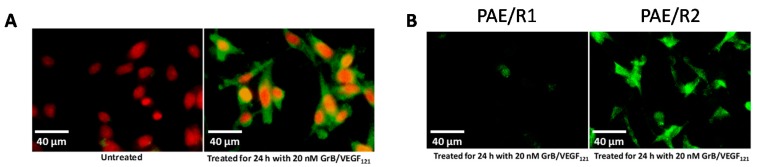
In vitro internalization of GrB/VEGF_121_ into target cells. (**A**) GrB/VEGF_121_ efficiently internalized into VEGFR-2-overexpressing endothelial cells within 24 h. (**B**) The efficiency by which GrB/VEGF_121_ internalizes into VEGFR-2-overexpressing endothelial cells over VEGFR-1-overexpressing cells.

To ensure that toxicity of GrB/VEGF_121_ is both targeted and specific, we evaluated a panel of endothelial and tumor cells lines with varying levels of VEGFR-1 and VEGFR-2 on their cell surface. Porcine cell lines over-expressing human VEGFR-1 or VEGFR-2 are considered to model tumor neovasculature, and have been used as in vitro models of angiogenesis [66]. Other cell lines were selected based on our previously established cytotoxic profiles with VEGF_121_/rGel, as well as cell surface VEGFR-1 and VEGFR-2 levels, as established by flow cytometry. In vitro cytotoxicity studies over 72 h showed varying levels of sensitivity to GrB/VEGF_121_ that correlated closely to total VEGFR-2 expression. IC_50_ levels were found to be in the nanomolar range (Figure 2A,B). The cytotoxicity was determined to be VEGFR-driven, as pre-incubation of VEGFR-2^+^ cells with 1 µM VEGF_121_ significantly reduced the cytotoxicity of GrB/VEGF_121_, but had no impact on the cytotoxicity of GrB alone (Figure 2C). In VEGFR-2 positive endothelial cells, GrB/VEGF_121_ showed rapid internalization into the entire cytoplasmic space within 24 h, while the internalization into VEGFR-1-expressing cells was significantly reduced (Figure 3), further confirming the VEGFR-driven targeting.

### 3.2. Pro-Apoptotic Activity of GrB/VEGF_121_

**Figure 4:**
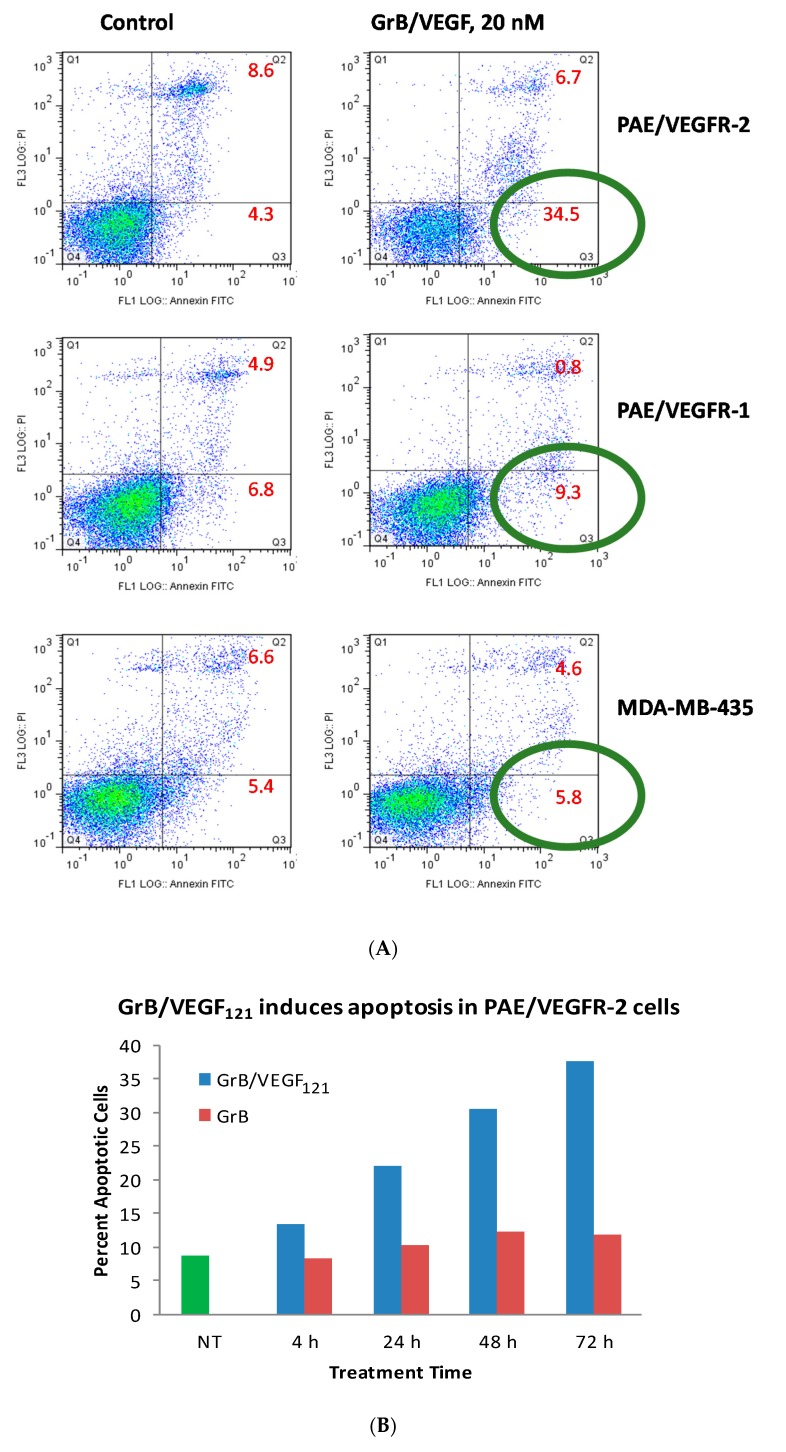
GrB/VEGF_121_ triggers apoptosis in target cells. (**A**) Annexin V/PI staining at 24 h after onset of incubation with GrB/VEGF_121_. Circles indicate the percent of cells in early apoptosis. (**B**) Induction of apoptosis over 72 h in VEGFR-2-overexpressing endothelial cells by the targeted GrB/VEGF_121_ compared to the non-targeted GrB. Green: untreated control; red: GrB treatment; blue: GrB/VEGF_121_ treatment.

While Granzyme B has well known caspase-dependent and independent mechanisms of apoptosis, it was important to examine whether the fusion protein itself elicited cytotoxicity by these pathways. We first evaluated the physical method of cell death of cells treated with GrB/VEGF_121_. Over a 24 h period, 35% of GrB/VEGF_121_-treated VEGFR-2^+^ cells mobilized into early apoptosis, compared to 4% of control cells. The impact on VEGFR-1^+^ endothelial cells and on the breast cancer cell line MDA-MB-231 cells was minimal (Figure 4A). When compared over a 72 h period, the percent of VEGFR-2^+^ endothelial cells undergoing apoptosis continued to increase compared to cells treated with Granzyme B alone (Figure 4B).

**Figure 5:**
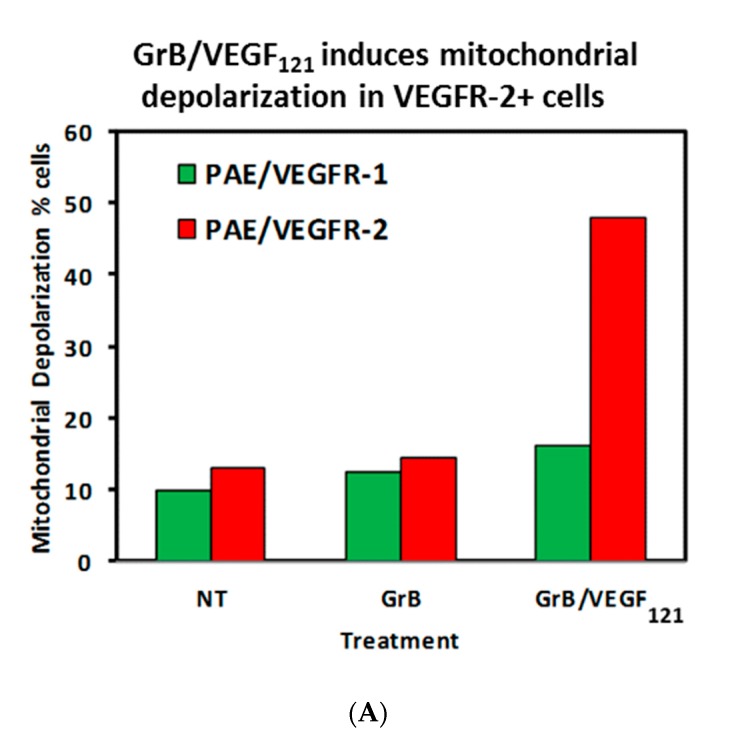

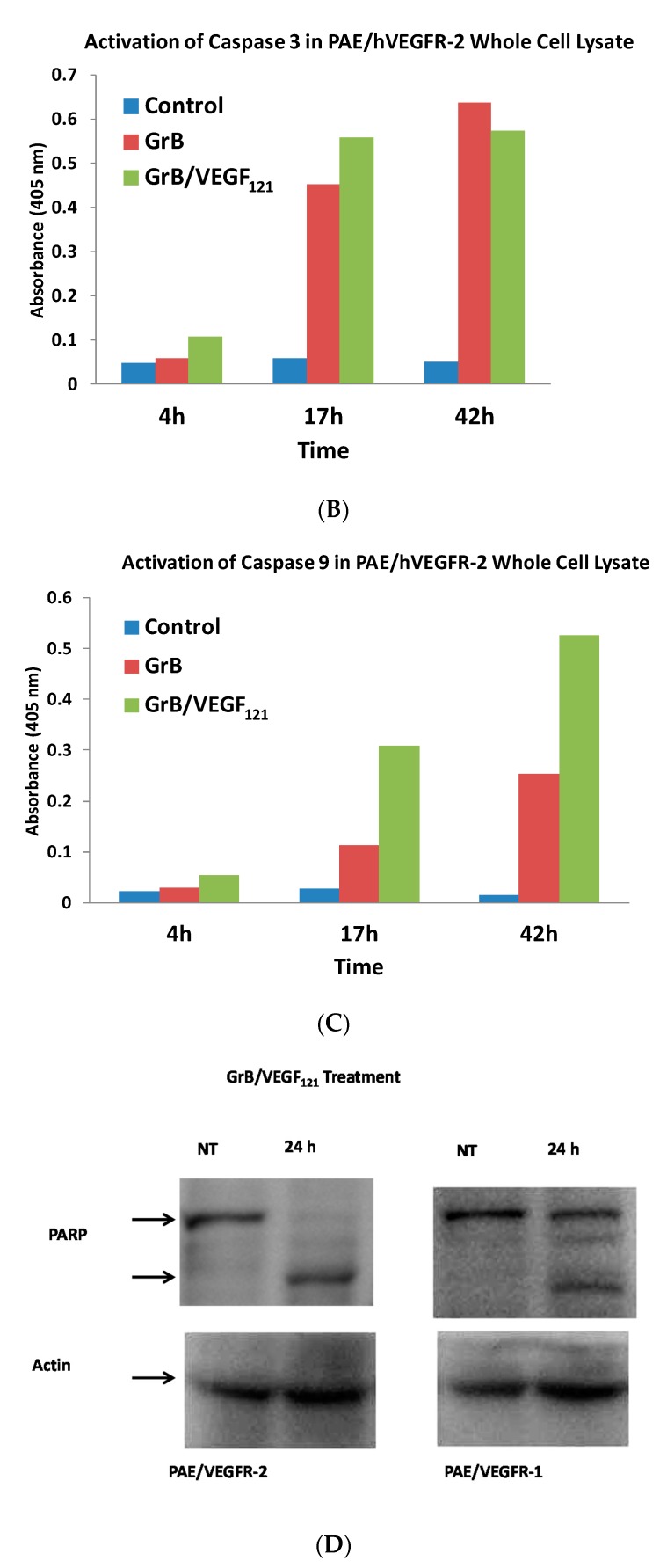
GrB/VEGF_121_ triggers mitochondrial depolarization and caspase activation in target cells, resulting in PARP-1 cleavage. (**A**) GrB/VEGF_121_ selectively triggered mitochondrial depolarization over 48 h selectively in VEGFR-2-overexpressing endothelial cells. (**B**) Using a Caspase-3 chromogenic substrate, Caspase-3 activation was also observed, as was (**C**) activation of Caspase-9 with the appropriate chromogenic substrate. (**D**) PARP cleavage was more robust and complete in VEGFR-2-overexpressing endothelial cells, compared to VEGFR-1-overexpressing endothelial cells.

To review whether apoptosis was triggered through well-characterized pathways, we analyzed the cell pathways by which apoptosis occurred. Forty percent of VEGFR-2^+^ cells underwent mitochondrial depolarization within 24 h of exposure to GrB/VEGF_121_, compared to 13% of controls, while VEGFR-1^+^ cells seemed largely unaffected (Figure 5A). Over this same time period, both Granzyme B and GrB/VEGF_121_ triggered caspase-3 activation in the VEGFR-2^+^ cells (Figure 5B). Caspase-9 activation was also observed, with GrB/VEGF_121_ apparently more efficient in activating this target than Granzyme B (Figure 5C). Finally, complete cleavage of Poly (ADP-ribose) polymerase-1 (PARP-1) was observed after 24h in VEGFR-2^+^ cells, whereas minimal cleavage was seen in VEGFR-1^+^ cells (Figure 5D). Notably, there was no demonstrated impact of a pan-caspase inhibitor on GrB/VEGF_121_-mediated cytotoxicity, while cleavage inhibition of both caspase-3 and caspase-9 showed. Thus, various downstream mediators of apoptosis have been shown to be engaged and activated upon treatment with GrB/VEGF_121_ in both a caspase-dependent and caspase-independent manner.

## 4. In Vivo Studies with GrB/VEGF_121_

### 4.1. Localization of GrB/VEGF_121_ into Tumor Tissue

**Figure 6:**
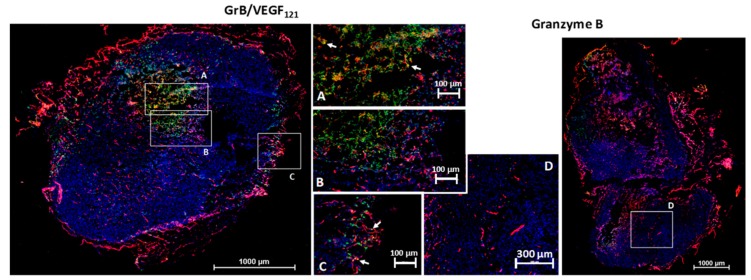
GrB/VEGF_121_ localizes into the tumor and tumor perivascular space. Sections were stained with immunofluorescence reagents to detect murine blood vessels (CD31, red), nuclei (Hoechst, blue) and Granzyme B (green). Co-localization of GrB into CD31^+^ tumor vessels appears yellow (representative areas indicated with white arrows). (**A**–**C**) Localization of GrB/VEGF_121_ into the PC-3 (prostate) tumor and the tumor perivascular space, as determined by immunofluorescence detection. (**D**) Free Granzyme B was not detected in the tumor using the same detection methods.

We investigated the ability of GrB/VEGF_121_ to localize into primary tumors by the subcutaneous placement of human prostate PC-3 tumors into male nude mice. This cell line is insensitive to VEGF_121_-mediated targeting in vitro, suggesting that it expresses insufficient receptor levels for targeted cytotoxicity. However, VEGF_121_-mediated targeting, in vivo with other cytotoxic payloads, has resulted in a significant anti-tumor therapeutic effect, presumably due to the highly vascularized nature of these tumors. Following tumor placement, mice were injected intravenously (tail vein) with GrB/VEGF_121_ or GrB at molar-equivalent doses. Four hours after administration, tissues were removed and snap frozen. Immunofluorescence staining revealed GrB/VEGF_121_ in tumor vessels as well as in in perivascular tumor areas adjacent to tumor microvessels (Figure 6). Normal organs did not show GrB/VEGF_121_ localization, nor did organs of mice treated with free Granzyme B, indicating the specific localization of GrB/VEGF_121_ into tumor tissue. Similar results have been observed with MDA-MB-231/Luc tumors [67].

### 4.2. In Vivo Efficacy of GrB/VEGF_121_

**Figure 7:**
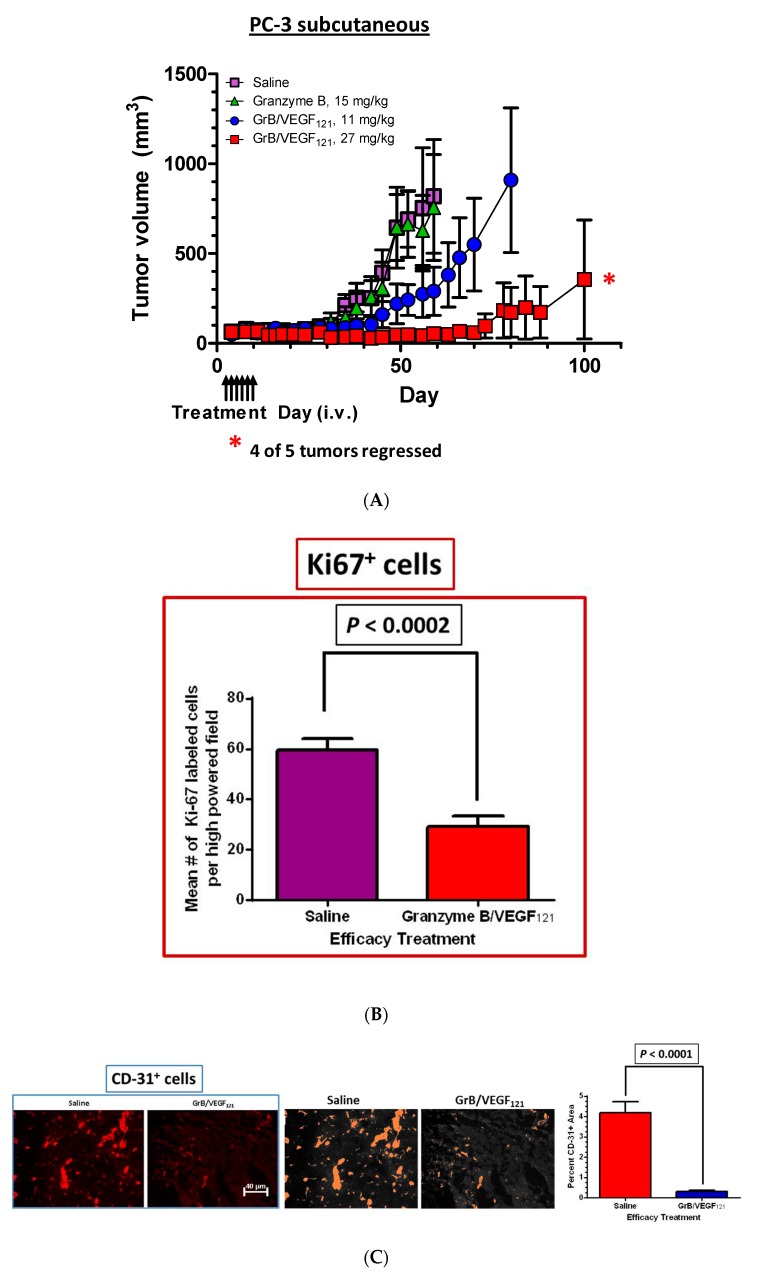
GrB/VEGF_121_ causes regression in established tumors and a reduction in tumor proliferation. Once tumors were measurable, mice received intravenous injections of either saline, GrB (15 mg/kg) or GrB/VEGF_121_ (11 or 27 mg/kg). (**A**) Treatment with GrB/VEGF_121_ caused a significant reduction in the growth of the primary tumor in nude mice. i.v.: intravenous injection. (**B**) The reduction in tumor growth is partially attributed to the reduction in the proliferation rate of the tumor cells. (**C**) In addition, tumor vasculature was significantly reduced upon GrB/VEGF_121_ treatment, with a greater than 14-fold reduction compared to mice treated with saline.

The efficacy of GrB/VEGF_121_ was also evaluated in a PC-3 subcutaneous tumor model. Mice received intravenous injections of saline, Granzyme B or GrB/VEGF_121_ every other day for a total of six treatments, and tumor volume was monitored twice weekly. Mice treated with GrB/VEGF_121_ at 27 mg/mg had very limited tumor growth, whereas those treated with a lower dose of GrB/VEGF_121_ (11 mg/kg) showed tumor growth of about threefold over 30 days. In contrast, tumors of saline- or Granzyme B-treated mice grew over 15-fold over the same period of time (Figure 7A). The reduced growth rate of GrB/VEGF_121_-treated mice compared to vehicle was confirmed by determining the number of cycling tumor cells in lesions, and found to be reduced by over 50% (Figure 7B). The impact of tumor vasculature was assessed by histopathologic analysis of the vascular area, and found to be significantly lower in GrB-VEGF_121_-treated mice compared to controls (Figure 7C).

## 5. Conclusions

The serine protease GrB appears to be an ideal payload for targeted therapy, in part because GrB exerts a multimodal and well-known mechanism of cytotoxic action. Fusion constructs targeting tumor cells or tumor vasculature and containing GrB are generally active in the low nanomolar range, and studies in tumor xenograft models demonstrate excellent antitumor efficacy. Concerns regarding immunogenicity should be mitigated by designing completely human constructs containing GrB, and the lack of toxicity in animal model studies of GrB-based fusion proteins thus far is encouraging. Expression of VEGFR on the cell surface of other potential targets, such as bone marrow-derived cells that are involved in local or distant tumor development, suggest that this construct may have promise in a multi-targeted approach. Indeed, the potential of other VEGFR-targeting constructs in inhibiting tumor growth in this manner has previously been demonstrated [28]. In addition, the relative lack of systemic toxicity of this agent points to potential utility in combination therapy with standard chemotherapeutic agents. Overall, Granzyme B delivered directly to tumor cells or tumor neovasculature appears to be a promising and exciting opportunity in the field of targeted therapy, with significant therapeutic potential in primary and metastatic disease.

## Supplementary Materials

The following are available online at www.mdpi.com/2227-9059/5/3/42/s1.
